# Multifaceted Role of Specialized Neuropeptide-Intensive Neurons on the Selective Vulnerability to Alzheimer’s Disease in the Human Brain

**DOI:** 10.3390/biom14121518

**Published:** 2024-11-27

**Authors:** Manci Li, Nicole Flack, Peter A. Larsen

**Affiliations:** 1 Department of Electrical and Computer Engineering, College of Science and Engineering, University of Minnesota, Minneapolis, MN 55455, USA; 2 Department of Veterinary and Biomedical Sciences, College of Veterinary Medicine, University of Minnesota, St. Paul, MN 55108, USA; 3 Minnesota Center for Prion Research and Outreach, College of Veterinary Medicine, University of Minnesota, St. Paul, MN 55108, USA

**Keywords:** single-cell gene expression analysis, RNA-seq, dementia, Alzheimer disease, neuropeptides, neurons

## Abstract

Regarding Alzheimer’s disease (AD), specific neuronal populations and brain regions exhibit selective vulnerability. Understanding the basis of this selective neuronal and regional vulnerability is essential to elucidate the molecular mechanisms underlying AD pathology. However, progress in this area is currently hindered by the incomplete understanding of the intricate functional and spatial diversity of neuronal subtypes in the human brain. Previous studies have demonstrated that neuronal subpopulations with high neuropeptide (NP) co-expression are disproportionately absent in the entorhinal cortex of AD brains at the single-cell level, and there is a significant decline in hippocampal NP expression in naturally aging human brains. Given the role of NPs in neuroprotection and the maintenance of microenvironments, we hypothesize that neurons expressing higher levels of NPs (HNP neurons) possess unique functional characteristics that predispose them to cellular abnormalities, which can manifest as degeneration in AD with aging. To test this hypothesis, multiscale and spatiotemporal transcriptome data from ~1900 human brain samples were analyzed using publicly available datasets. The results indicate that HNP neurons experienced greater metabolic burden and were more prone to protein misfolding. The observed decrease in neuronal abundance during stages associated with a higher risk of AD, coupled with the age-related decline in the expression of AD-associated neuropeptides (ADNPs), provides temporal evidence supporting the role of NPs in the progression of AD. Additionally, the localization of ADNP-producing HNP neurons in AD-associated brain regions provides neuroanatomical support for the concept that cellular/neuronal composition is a key factor in regional AD vulnerability. This study offers novel insights into the molecular and cellular basis of selective neuronal and regional vulnerability to AD in human brains.

## 1. Introduction

Alzheimer’s disease (AD) is a neurodegenerative disorder characterized by progressive cognitive decline and memory loss [1]. The neuropathological features of AD encompass both “positive”—Aβ plaques and tau tangles, glial responses, and cerebral amyloid angiopathy—and “negative” lesions, such as the loss of neurons and synapses [2]. The progression of AD pathology in the brain follows a stereotypical pattern (Braak stages): In the early stages of AD, the transentorhinal regions are primarily affected, followed by the spread of pathology to neocortical regions in later stages [3]. The majority of AD cases occur sporadically, with no clear understanding of their cause or pathogenesis [1]. However, aging is the biggest risk factor for developing AD. The decline in cognitive function during natural aging bears resemblance to that seen in the early stages of AD [4,5]. Mild cognitive impairment (MCI) is defined as a stage between normal age-related cognitive changes and pathological cognitive impairments, and early AD is frequently preceded by MCI [6].

“Epicenters” are described as the sites exhibiting the peak pathological changes or atrophy within the brain, often considered to coincide with the initial site of disease onset by the network-based degeneration/spread hypothesis for AD [7,8]. Such regions, including the entorhinal cortex (EC), could also be interpreted as brain regions that are selectively vulnerable to AD [9]. Despite extensive research, the mechanisms underlying the selective vulnerability of neuronal subtypes and brain regions to cellular dysfunction and protein misfolding are unknown [9,10]. Investigating “epicenters” from the perspectives of cellular processes, the temporal progression of AD and aging, and the spatial vulnerability of brain regions to AD could provide valuable insights into this enigma.

The susceptibility of a particular brain region to certain diseases may be influenced by the inherent vulnerabilities of the specific cell types and states found within that region [9,11]. Single-cell sequencing technology has revolutionized our understanding of neuronal diversity by revealing a large number of neuronal subtypes that extend beyond the previously established categories [12], which were generally defined using molecular markers in combination with morphology and other cellular characteristics [13,14,15]. The expression of neuropeptides (NPs) has played a pivotal role in neuronal heterogeneity by assisting single-cell transcriptomic neurotaxonomy—an approach first introduced by Tasic and then applied by Smith et al. in their study of mouse brains as a proof of concept [16,17]. Subsequently, the comprehensive single-cell transcriptomic investigation of adult human brains also found that neuronal subpopulations can have complex and combinatorial NP co-expression networks, many of which are uniquely localized to specific brain regions [12]. Therefore, single-cell transcriptomic investigation of NPs offers a unique opportunity to elucidate the vulnerability of brain regions based on the susceptibility of the specific neuronal populations residing within them, particularly in the context of AD.

Beyond the role of neuronal identity, the neuroprotective and homeostatic functions of NPs may be of even greater value in understanding and treating AD. Adding to previous reports on NP dysfunction in AD [18], we recently reported a widespread disruption of NP networks and a disproportionate absence of neurons with high NP expression in the entorhinal cortex of AD brains [19]. These findings were further corroborated by subsequent research, highlighting the involvement of NPs in AD neuropathology and neurodegeneration during aging [20,21]. Given the crucial role of NPs in intercellular communication and neuronal health [18], they could have a significant impact on the earlier stages and potentially the etiology of AD. As NPs act through G protein-coupled receptors (GPCRs), which are among the most druggable targets for treating diseases in the central nervous system [22], investigating the role of NPs in the selective vulnerability of AD could be fruitful for developing preventative strategies and targeted interventions for AD, especially considering that GPCRs did not exhibit changes as significant as those of NPs in AD brains [19].

**Figure 1:**
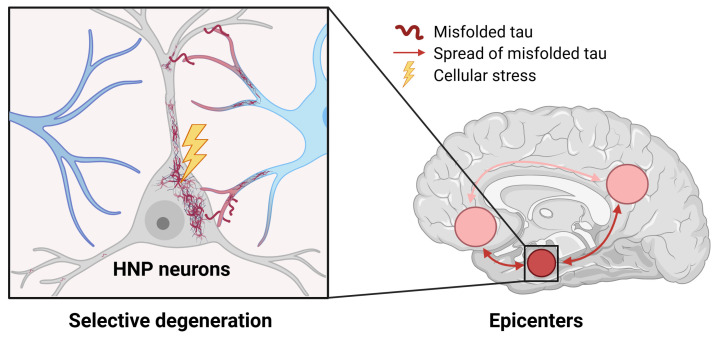
Hypothetical model illustrating the potential mechanisms underlying the selective vulnerability of high-neuropeptide-producing (HNP) cells and their contribution to region-specific emergence of Alzheimer’s disease (AD). The model proposes that the unique functions of HNP neurons make them more prone to stress and protein misfolding, and this susceptibility becomes more evident with advancing age. The regional vulnerability observed in Alzheimer’s disease can be attributed to the distribution and density of these cells, as well as their excretory functions and interactions with other brain networks. Specifically, it predicts that (1) temporal, limbic, and prefrontal cortical regions have a higher density of HNP neurons expressing ADNPs; (2) disruption of cellular processes in HNP neurons leads to a decrease in ADNPs during aging and localized formation of misfolded proteins in AD, causing various degrees of cognitive decline and selective degeneration of these neurons; and (3) the dynamic paracrine and secretory activities of HNP cells facilitate the propagation of misfolded proteins and transneuronal degeneration in closely connected temporal, limbic, and prefrontal cortical regions, resulting in the widespread deposition of misfolded tau proteins in AD.

Considering the metabolic alterations in early AD and the potential energetic burden imposed on neurons with high levels of NP production (HNP neurons) [23,24], we hypothesize that regional dysfunction in AD may originate from these specialized neurons serving as focal points in the “epicenters” of cascading cellular dysfunction, including metabolic stress and protein misfolding (Figure 1) [8,9,10]. Cells expressing Alzheimer’s-associated NPs (ADNPs, Table S1) are expected to be particularly vulnerable. To test this hypothesis from the perspectives of cellular mechanisms (function), the continuum of AD progression and aging (time), and regional brain vulnerability to AD (space), we analyzed publicly available single-cell and spatiotemporal RNA-seq datasets, encompassing ~1900 human brain samples. We expect that: (1) HNP neurons will express enhanced functional networks, such as increased metabolic demands and protein misfolding vulnerability, that can contribute to AD development; (2) the abundance of HNP neurons, especially those co-expressing ADNPs, will decrease with AD progression; (3) the decrease in ADNP expression with aging will be more pronounced in early AD-impacted brain regions compared to regions affected later in the disease; and (4) HNP neurons co-expressing ADNPs will be preferentially distributed in the “epicenters” of AD, and their spatial pattern will coincide with the regional progression of AD pathology. If any of these expectations are not observed, our hypothesis should be revised.

## 2. Methods

### 2.1. Overview of Datasets and Analyses

**Figure 2:**
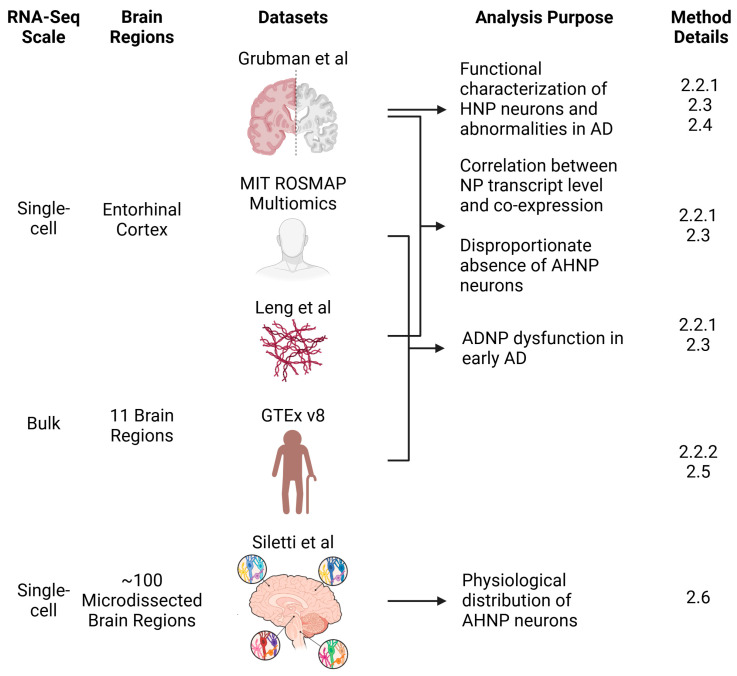
Schematic overview of datasets and analyses. Three single-cell RNA-sequencing datasets of the human entorhinal cortex (Grubman [25], Leng [26], and MIT ROSMAP Multiomics) were used to investigate the relationship between neuropeptide (NP) co-expression and Alzheimer’s disease (AD) progression. The Grubman dataset was used for mechanistic analyses, while the MIT ROSMAP Multiomics and Leng datasets were included in demonstrating the early involvement of Alzheimer’s disease-associated neuropeptides (ADNPs) during AD development/progression over time from the aspect of cognitive status and neuropathology. Bulk transcriptomic data from the GTEx project were used to study the expression of ADNPs during aging. A comprehensive description of neurons co-expressing high levels of ADNPs (AHNP neurons) across microdissected brain regions was performed using the Siletti et al. [12] single-cell dataset. The method details indicate where the analysis information can be found in the Methods section. Sing-cell, single-cell RNA-sequencing; Bulk, bulk RNA-sequencing; HNP neurons, high NP-expressing neurons. The arrows and brackets indicate the datasets used for each purpose.

A schematic overview of the analytical workflow is presented in Figure 2. Code for the bioinformatic analyses included in this study can be found in Supplementary Materials.

Three publicly available single-cell RNA-sequencing datasets of the human EC were included for analysis in this study: Grubman et al. (the Grubman dataset) included samples from 6 control and 6 AD brains [25]. Leng et al. (the Leng dataset) focused on progression of neuropathology in AD, sampling three donors from Braak stage 0, four donors from Braak stage 2, and three donors from Braak stage 6 [26]. The EC dataset associated with Mathys et al.—generated by the MIT ROSMAP Single-Nucleus Multiomics Study (the MIT ROSMAP Multiomics dataset)—centered on the cognitive status of donors [21,27]; we included 8 control, 8 MCI, and 8 AD samples. To establish that a higher number of co-expressed NPs can serve as a proxy marker for HNP neurons, based on the premise that higher NP co-expression is indicative of greater NP transcript levels [19], correlational analyses were performed using three single-cell RNA-sequencing datasets. The method was motivated by the following reasons: (1) the dataset from the Siletti et al. study (detailed below) represents a robust test of the hypothesis at the spatial level, providing insights into the selective vulnerability of neuronal subpopulations and brain regions in AD based on “combinatorial neuropeptide co-expression” [12]; (2) the presence or absence of a gene is likely more conserved across different studies than exact transcript counts; and (3) this approach facilitates future empirical studies investigating HNP neurons.

The distribution of neurons based on neuropeptide (NP) co-expression was assessed for each dataset. Only the Grubman et al. dataset exhibited a distribution similar to those previously reported in high-quality mouse RNA-seq datasets [17], making it suitable for the mechanistic portion of this study—see below for detailed methods for differential gene expression, functional enrichment analysis, and regression analyses, as well as a hypergeometric test. Both the Leng and MIT ROSMAP Multiomics datasets had over 70% of neurons not expressing any NPs in the NP list compiled previously (Figure S1), which is significantly lower than expected [12,17,19]. Therefore, the ADNP list previously identified from the Grubman dataset was used throughout this manuscript [17,19].

Despite the limitation of NP expression, the Leng and MIT ROSMAP Multiomics datasets were included in the analysis of AD development and progression (time), as they characterize changes of neuropathology and cognitive functions during AD pathogenesis. The Genotype-Tissue Expression (GTEx) project’s v8 data release included bulk transcriptomic data derived from tissues sampled from donors aged 20 to 79 years [28], providing a valuable resource for studying gene expression changes during aging [29,30,31]. All brain regions from the GTEx v8 data were included for this study, subject to further exclusion criteria applied to ensure data quality (See below).

A comprehensive analysis of ADNP occurrences and cell counts across microdissected brain regions was performed using the single-cell transcriptomic dataset generated by Siletti et al. [12]. The dataset comprised samples from 3 postmortem human brain donors, encompassing approximately 100 microdissected regions, 2 million neurons, and 461 clustered neuronal subpopulations. The dataset was accompanied by an annotated cell cluster (cluster_annotation) file that tagged clustered neuronal subpopulations with co-expressed NPs [12], which was used in this study.

### 2.2. Acquisition and Preprocessing of Transcriptome Data

#### 2.2.1. Single-Cell RNA-Seq Data from the Human Entorhinal Cortex (EC)

Data generated by Grubman et al. from 6 control (CT) and 6 AD donors (12 total) were obtained [25]. The detailed documentation of data acquisition, preprocessing steps, and downstream analyses (dimension reduction and cell identification) can be found in Li and Larsen (2023) [19]. Briefly, cells were filtered based on gene expression and mitochondrial content, retaining those with 200–2500 expressed genes and less than 5% mitochondrial reads. Data normalization followed Seurat’s (version 4.1.1; R 4.2.3 unless specified) guidelines using a count per million (CPM) matrix [32]. Using cell identification methods from *BRETIGEA* and Grubman et al., six primary cell types were identified: astrocytes, microglia, neurons, oligodendrocytes, oligodendrocyte precursor cells, and endothelial cells. Cells were labeled by their highest association score, but some were categorized as unidentified or hybrid based on specific criteria described in detail by Grubman et al. as well as Li and Larsen [19,25]. Only cells identified as neurons were used in the downstream analyses.

Preprocessed and annotated single-cell expression matrices and metadata from the Leng et al. study were obtained from CZ CELLxGENE [33]. Cells annotated as excitatory and inhibitory neurons from the entorhinal cortex were included for downstream analyses.

Preprocessed and annotated single-cell expression matrices and metadata from the MIT ROSMAP Multiomics study were downloaded from ADKnowledge portal [21,34]. Three diagnostic categories from the ROSMAP study were included in the presented study: control (CT or NCI: no cognitive impairment), mild cognitive impairment (MCI: no other condition contributing to CI), and AD (Alzheimer’s dementia: no other condition contributing to CI (NINCDS/ADRDA Probable AD)) [27]. The clinical study design and detailed diagnostic criteria were described in the original ROSMAP manuscript and deposited on the ADKnowledge portal [6,34]. Individuals with inconsistent clinical diagnosis, clinical cognitive diagnosis summary, and final consensus cognitive diagnosis documented in the ROSMAP study were excluded from the analysis. Methods for isolation of nuclei from frozen post-mortem brain tissue, droplet-based snRNA-seq, and snRNA-seq data preprocessing are available in detail on the ADKnowledge portal [27]. De-identified metadata for individuals and experiments included in this study were detailed in supplementary materials (Table S2). To provide equal representation of each condition, we randomly selected eight AD and CT samples (set.seed = 123) to match the eight available MCI samples. Overall, 41,373 neurons (n_neuron = 41,373) from eight AD (n_neuron = 16,214), MCI (n_neuron = 11,732), and CT (n_neuron = 13,427) EC regions were included in the final analysis.

#### 2.2.2. Spatiotemporal Bulk RNA-Seq Data from Human Brains

RNA-seq transcript matrices (transcript per million (TPM)) of 11 human brain regions from a cohort of individuals from the general population were obtained from the GTEx portal (Table S3) [35]. Samples from individuals that lacked complete metadata regarding age, sex, or death classification were excluded, as were those that scored 3 or 4 on the Hardy scale that indicates intermediate or slow death. Only samples with an RNA integrity number (RIN) larger than 6 were included in the analysis. The results were visualized with cerebroViz (version 1.0; R 3.6.3) [36] and assembled in BioRender.

### 2.3. Single-Cell Correlational Analysis of NP Transcripts and NP Co-Expression

The Spearman rank correlation was utilized to assess the relationship between the transcript level of NP and the number of co-expressed NPs for all human single-cell transcriptome datasets (i.e., the Grubman, Leng, and MIT ROSMAP Multiomics datasets) using *cor.test* in R [21,25,26,34]. The correlation coefficient (rho) and *p*-values were reported. The significance cut-off was set at 0.05.

### 2.4. Downstream Analyses for Single-Cell Transcriptome Data from Human EC

#### 2.4.1. Differential Gene Expression (DGE) Analysis

As such, DGE analysis was performed for the single-cell dataset generated by Grubman et al. [25]. The number of co-expressed NPs was used as a proxy for transcript levels of NPs; neurons were divided into low (0–1), medium (2–5), and high (6+) NP-producing groups (LNP, MNP, and HNP) based on the number of co-expressed NPs in both conditions. DGE analysis was implemented between LNP and HNP in control neurons as well as MNP groups in control and AD using the *FindMarkers* function in *Seurat* [32]. Default parameters for DGE in *Seurat* were used (Wilcoxon rank sum test); the statistical cut-off was set at 0.05 for a false discovery rate (FDR) adjusted by the Benjamin–Hochberg (BH) method. Pseudo-bulk methods were not applied, as they would lead to reduced statistical power [37]; given the relatively small sample size and number of cells in the Grubman dataset, using pseudo-bulk would limit our ability to detect biologically meaningful differences in gene expression.

#### 2.4.2. Functional Enrichment Analysis

The output of the DGE analysis from *Seurat* was used as input to the STRING database (version 11.5) [38]. Key enrichment output from STRING analysis was visualized using the *Enrichplot* package (version 1.18.4) [39]. STRING uses the Bonferroni method to correct for multiple comparisons and provides adjusted *p*-values [38]. The significance cut-off was set at 0.05 for FDR.

#### 2.4.3. Regression Analysis of NP Transcripts and ADNPs Co-Expression

Regression analysis was used to discern the relationship between gene transcript levels and the presence of NP. Utilizing the *glm* function in R, general linear models were constructed for each differentially expressed gene (DEG), with transcript levels as the response variable and the number of co-expressed NPs as the explanatory variable. The BH method was used to adjust for multiple comparisons for all *p*-values; 0.05 was used as the significance cut-off for adjusted *p*-values. The goal was to identify genes whose expression is notably influenced by the increased number of co-expressed NPs.

#### 2.4.4. Hypergeometric Test of Cell Abundance and ADNP Co-Expression

The hypergeometric test was employed using the *phyper* function in R to assess the overlap of genes with decreased expression in AD compared to those that are functionally enriched in HNP neurons [40]. Specifically, the following were defined: (1) the number of overlapped genes as “successes” in our sample (x = 25); (2) genes with significantly decreased expression in AD MNP neurons as “successes” in the population (m = 91); (3) the total number of unique genes expressed by AD MNP and control HNP neurons, minus those with decreased expression in AD MNP neurons, as “failures” in the population (n = 19,430); and (4) genes with significantly increased expression in HNP neurons as the sample size (k = 307). Because *phyper* is a cumulative distribution function, the conduction of a one-tailed such analysis (*lower.tail = FALSE*) would calculate a probability of observing as extreme and more extreme results in the direction of higher values (*p*-value) [40]. The significance cut-off was set at 0.05.

### 2.5. Spatiotemporal Correlation Analysis Between ADNP Gene Expression and Age in the Human Brain

In this study, aging is proxied by pseudo-aging, defined as a cross-sectional approach that simulates the effects of aging by analyzing samples from individuals of varying ages at the time of death. This method enables the assessment of age-related changes at a single point in time, addressing ethical concerns and the practical impossibility of longitudinally collecting brain tissues from multiple regions in human subjects.

Gene lists of NPs and ADNPs were downloaded from existing publications [19,41]. The TPM count matrix of spatiotemporal RNA-seq data and metadata of human brains were downloaded from GTEx [35]. All TPM counts were log-transformed. The following exclusion criteria were applied to ensure data quality for the analysis. First, individuals lacking complete metadata, including age, sex, and death classification, were removed from the dataset. Second, to ensure only high-quality RNA samples were used, subjects with Hardy scores of 3 or 4, indicating intermediate or slow death, and those with RNA integrity numbers (RIN) lower than 6, were excluded from the analysis. Finally, as brain development is known to continue throughout the early 20s [42], subjects aged 20 to 29 years were excluded to focus on age-related changes in the mature brain. The relationship between the expression of individual ADNPs and their cumulative expression with respect to age was investigated using the *PResiduals* package (*megabot* function; version 1.0-1) [43], adjusting for RIN and Hardy scale. The significance cut-off was set at 0.05. Analyzing the cumulative expression of ADNPs, in addition to individual NPs, provides a biologically relevant (overall burden and decreased population of neurons/cells co-expressing them) and robust measure (combined effect of multiple ADNPs) of their changes collectively during aging, while also improving the signal-to-noise ratio.

### 2.6. Examination of ADNP-Co-Expressing HNP (AHNP) Neurons Across Microdissected Brain Regions

The file used in this study was downloaded from the GitHub link provided by Siletti et al. [12]. Firstly, the presence of ADNPs from the file was quantified, and counts for NPs were generated further to calculate non-ADNPs. Based on these counts, we defined ADNP-HNP (AHNP) neurons as those tagged with 6+ ADNPs and <3 non-ANDPs, accounting for the difference in the input NPs [12,19]. Concurrently, we estimated the number of cells in different brain regions and dissections based on percentage data extracted from the *cluster_annotation* file. These estimates were summed across unique regions and dissections to provide a granular view of cell distribution. Additional analyses were conducted on specific regions for MEC, where the dataset was grouped by various attributes such as neurotransmitter, subtype, and MTG label [44], and the number of cells in each group was summed and visualized.

## 3. Results

### 3.1. Alterations of HNP Neuronal Abundance and Functions in AD: Overlap of HNP Dysfunction and Molecular Signature of AD

**Figure 3:**
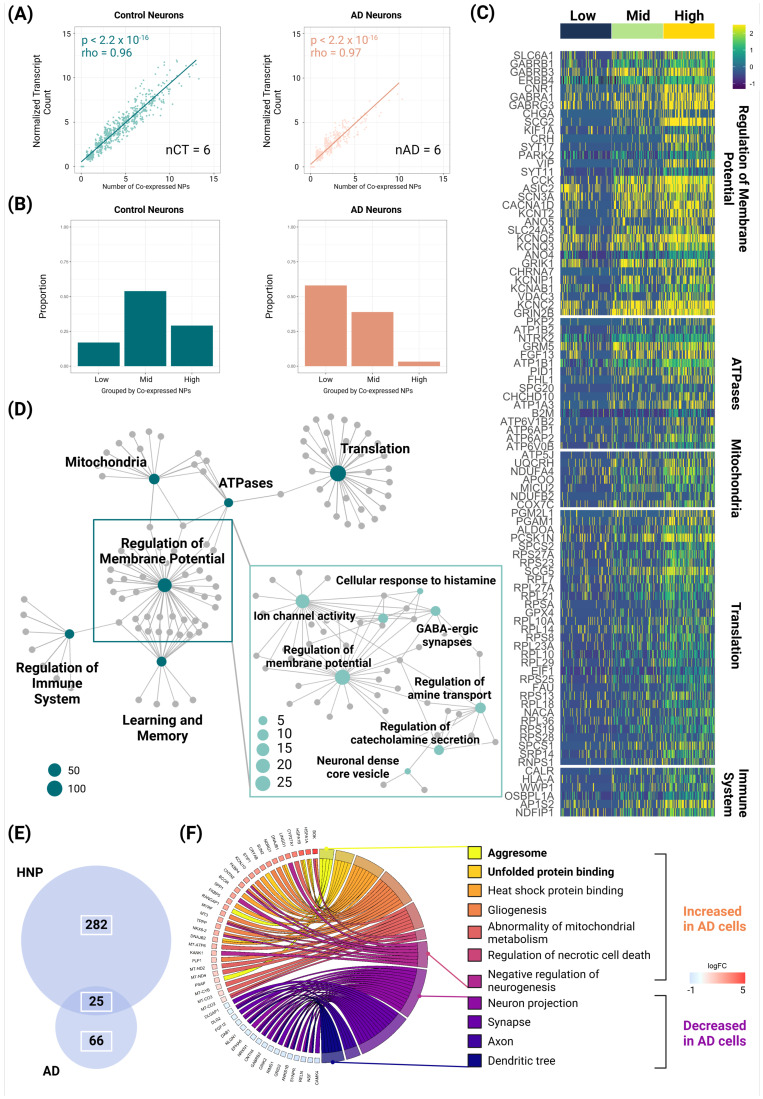
Changes in high-neuropeptide-producing (HNP) neuronal abundance and function: overlap of dysfunction and AD molecular signatures. (**A**) The relationship between transcript abundance and the number of co-expressed neuropeptides (NPs) in neurons from both control and AD EC. (**B**) The distribution of neurons based on the number of co-expressed NPs: low: 0–1; middle (mid): 2–5, and high: 6+. Proportion = in-group neuron counts in the condition/total neuron count in the condition. (**C**) Heat plot showing differentially expressed genes (increased) in HNP neurons (neurons in high NP co-expression group). (**D**) Gene network plot showing results of functional enrichment for HNP neurons. (**E**) Venn diagram showing the overlap between gene expressions higher in HNP neurons (neurons in high NP co-expression group that express 6+ NPs) and significantly decreased in AD neurons co-expressing 2–5 NPs (MNP neurons). The hypergeometric test was applied to evaluate the overrepresentation of genes upregulated in control HNP neurons but notably reduced in AD MNP neurons; *p* < 0.00001; α = 0.05. (**F**) Molecular signatures differentially increased and decreased in AD MNP neurons. nCT, number of healthy donors; nAD, number of AD donors.

Analyzing the single-cell dataset by Grubman et al. [19,25,45], we showed a very strong correlation (>96%) between transcript abundance and the number of co-expressed NPs generally exists for neurons in both control and AD groups (Figure 3A). This effect was also observed (>90%) in both the Leng and MIT ROSMAP Multiomics datasets (Figure S2). Applying the number of co-expressed NPs as a proxy for transcript levels of NPs, we stratified neurons into low (0–1), medium (2–5), and high (6+) NP groups. We observed a similar absence of HNP neurons in AD, which was also corroborated by the Leng and MIT ROSMAP Multiomics datasets (Figure 3B; Table S4; Figure S3) [19]. As explained in the Methods section, only the Grubman dataset was used for the following analyses.

As we hypothesized that HNP neurons would experience greater energetic demands and higher metabolic stress, we predicted that HNP neurons would show more metabolic activity. To test this, differentially expressed genes (DEG) were examined between high and low groups of control neurons followed by enrichment analysis [19,38]. We found that all the NPs expressed at significantly higher levels in HNP neurons were ADNPs [19], and genes required for NP transportation, translation, and metabolic processes were significantly increased in HNP neurons in comparison to LNP neurons (Figure 3C,D; Tables S1, S5 and S6). Although it is widely known that GABA is often co-expressed with NPs [46], we observed that HNP neurons also participated in other chemical communications, such as using histamine and catecholamines, to regulate membrane potential (Figure 3D; Table S5). In addition, ~36% of DEGs regulating membrane potentials were functionally enriched for learning and memory (Figure 3D; Table S5). Surprisingly, the regulation of innate immune response was increased in HNP cells (Figure 3C,D; Table S5).

To elucidate the increase observed across the LNP, MNP, and HNP groups, we sought to identify genes whose expression was significantly influenced by the level of NP co-expression, as a proxy for the abundance of NP transcripts. We utilized regression models to examine the relationship between gene expression and NP co-expression and then ranked the increased DEGs in HNP neurons by coefficients and R^2^. Excluding NP components, genes ranked in the top 10 for coefficients or R^2^ included sodium/potassium-transporting ATPase [47] and intracellular transport vesicles [48], supporting the hypothesized functional enhancement of HNP neurons (Table S7). Notably, *ERBB4*, the protein products of which induce tau hyperphosphorylation [49], was among the top genes related to NP co-expression (Table S7; Figure S4).

Because we discovered that HNP neurons exhibited higher performance in several expected cellular functions—including transportation, translation, and metabolic processes—and participated more heavily in other chemical communications, regulation of innate immune response, and circadian rhythm, we wondered whether dysregulation of processes in these functions may lead to the loss of neuronal functions and the accumulation of tau pathology, which are hallmarks of several neurodegenerative disorders including AD.

Speculating that disrupted functions of neurons expressing more NPs are associated with protein misfolding, we examined the DEGs for neurons stratified by the number of co-expressed NPs and analyzed the neurons in the medium group (as HNP neurons were virtually absent in AD brains). Enrichment analysis revealed that these cells displayed molecular characteristics related to both “positive” and “negative” neuropathology in AD [2]. We note the decreased DEGs in AD cells were significantly enriched for those functionally increased in HNP cells (*p* < 0.00001; Figure 3E; Tables S6 and S8), indicating that loss of HNP functions participates in the molecular pathogenesis of AD. Genes with protein products showing significantly decreased expression included those with functional roles in axons, synapses, and dendrites (Figure 3F; Table S8). Increased molecular processes included those known to be disturbed in AD, such as negative regulation of neurogenesis, gliogenesis, and abnormal mitochondrial metabolism (Figure 3F; Table S9). Notably, genes involved in forming aggresomes and unfolded protein binding were highlighted [50], indicating the active occurrence of protein misfolding in AD cells that co-express NPs (Figure 3F; Table S9).

### 3.2. ADNP Dysfunction Observed in Early Pathogenesis of AD

**Figure 4:**
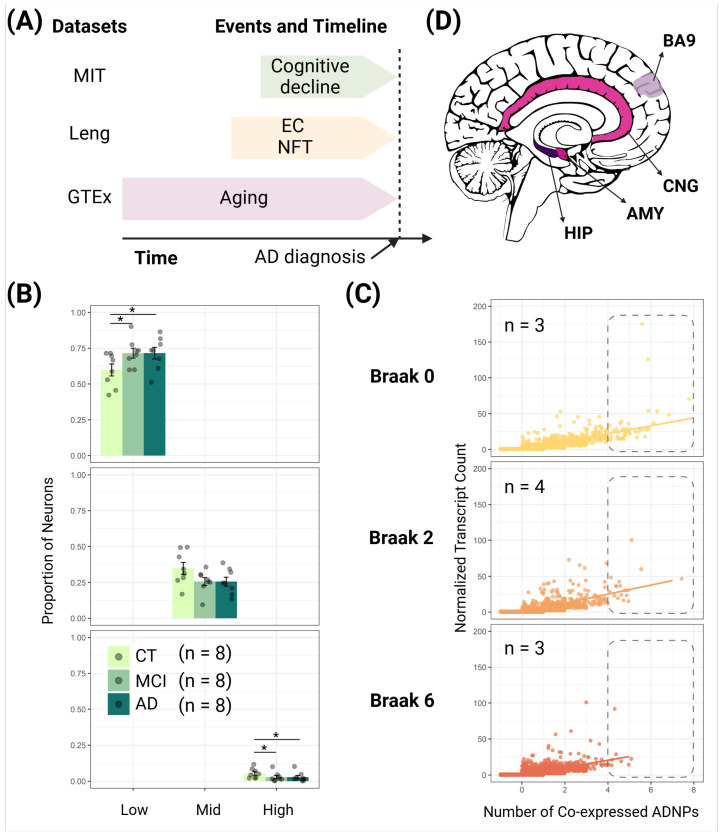
Decreased expression of Alzheimer’s disease-associated neuropeptides (ADNPs) across brain regions during disease progression and aging. (**A**) Timeline of datasets relative to typical diagnosis of AD. (**B**) Comparison of proportions of neurons from the entorhinal cortex (EC) of control (CT), mild cognitive impairment (MCI), and AD donor brains (MIT, MIT ROSMAP Multiomics dataset), stratified by the number of co-expressed ADNPs (Low: 0–1, Mid: 2–5, High, 6+). One-tailed Wilcoxon rank sum test was used. α = 0.05. * *p* < 0.05. (**C**) Scatterplot showing the relationship between transcript abundance and the number of ADNPs in neurons from donor brains classified as Braak stages 0, 2, and 6. The reduced abundance of neurons co-expressing higher levels of ADNPs during AD progression is lighted. (**D**) Brain regions (medial sagittal view) showing significant decrease in ADNPs with aging. AMY, amygdala; CNG, anterior cingulate cortex; HIP, hippocampus; BA9, Brodmann area 9; n, sample size.

To test the hypothesis that changes in the abundance of HNP neurons expressing ADNPs contribute to the selective vulnerability of brain regions and neuronal subpopulations in AD, we systematically analyzed three distinct datasets encompassing different stages and aspects of AD development. These datasets included: (1) the progression of cognitive impairment from normal aging to mild cognitive impairment (MCI) and finally to AD (the MIT ROSMAP Multiomics dataset) [21,51], (2) the advancement of AD neuropathology through different Braak stages (the Leng dataset) [26,33], and (3) the effects of aging, a major risk factor for AD, on ADNP expression in various brain regions (the GTEx v8 dataset) (Figure 4A) [28]. By integrating findings from these diverse datasets, we aimed to provide a comprehensive understanding of how changes in ADNP expression in HNP neurons relate to the spatiotemporal progression of AD pathogenesis. Given the disproportionate absence of neurons expressing ADNPs in the EC of AD brains and the higher level of ADNPs physiologically expressed by HNP neurons, we specifically focused on ADNPs in the following analyses.

Analysis of the MIT ROSMAP Multiomics dataset revealed that, despite the majority of neurons not expressing any NPs (Figure S1), patterns of the ADNP expression by neurons were similar to those observed in the Grubman dataset when considering cognitive status and AD. Specifically, both the MCI and AD groups had significantly more neurons co-expressing 0–1 ADNPs (CT vs. MCI *p*-value = 0.025; CT vs. AD *p*-value = 0.025) and significantly fewer neurons expressing 6+ ADNPs compared to cognitively normal individuals (CT vs. MCI *p*-value = 0.032; CT vs. AD *p*-value = 0.014) (Figure 4B). But they were not statistically different from each other at any point (Low MCI vs. AD *p*-value = 0.36; Mid MCI vs. AD *p*-value = 0.40; High MCI vs. AD *p*-value = 0.60) (Figure 4B). We note that most of the MCI donors (6/8) had a Braak stage of 1–2 (Table S2). Similarly, although the Leng dataset had even fewer neurons expressing NPs (Figure S2), results from visualizing the ADNP co-expression and transcript levels were consistent with the observations from the MIT ROSMAP Multiomics dataset, clearly demonstrating a depletion of HNP neurons expressing ADNPs in donors with Braak stages 2 and 6 (Figure 4C). These results provided further evidence that the loss of HNP neurons expressing ADNPs occurs early in the AD neuropathological process. Overall, it was surprising to find that as early as Braak stage 2, there was already a stark contrast between control and Braak 2 donors in ADNP expression patterns similar to those observed late in AD.

To investigate even earlier in the disease process and study the spatiotemporal changes of ADNPs in the context of aging, a major risk factor for AD, we analyzed bulk RNA-sequencing data generated by the GTEx consortium [28]. We expected that if HNP neurons expressing ADNPs diminish during the aging process, a corresponding decrease in ADNP expression levels would be observed in this dataset, particularly in brain regions affected early in AD. Our recent report indicated that ADNP expression decreased with age in the hippocampus [19]; however, we do not yet know if the decline of NPs with aging is ADNP- and brain region-specific. If our hypothesis were to hold, only brain regions affected by early AD should show age-related changes in ADNP expression, and only the accumulative expression of ADNPs, but not other NPs, should decrease with age in the human brain. We first examined the expression of ADNPs during aging among 11 brain regions selected from GTEx (Table 1) [19,35]. As expected, we found that only the hippocampus, frontal cortex, anterior cingulate gyrus, and amygdala—all of which are brain regions affected by early AD [52,53,54,55,56,57]—showed a significant decrease of ADNP transcription during aging among the 11 brain regions selected from GTEx (Table 1; Figure 4D) [3,19,35].

We also examined the expression of NPs in these brain regions and whether the expression of individual NPs was correlated with age. We found that NPs demonstrating a significant decrease in expression with age in the aforementioned brain regions consisted mostly of ADNPs (Tables S1 and S10) [19]. To support the specificity of the observed change in ADNP expression, we analyzed the transcript count of all non-ADNP NPs in the brain regions and found no age-related changes specific to AD-related regions in their overall levels (Table S11). We confirmed that the larger decrease of NP expression in these brain regions with aging was not attributed to their intrinsic capacity to express NPs: (1) Using this dataset and the NP list compiled previously, ~80 NPs were expressed by each brain region (Table S12); (2) the differences in the total NP transcript count among the surveyed brain regions do not explain the AD-specific patterns observed in Table 1 (Table S12). In short, the spatiotemporal transcriptomic analysis presented here showed that the decrease of NPs in the aging human brain was specific to brain regions and NPs implicated in AD, supporting the idea that age-related cognitive decline shares mechanisms with AD and may be mediated by loss of ADNP expression during aging.

### 3.3. Physiological Distribution of AHNP Neurons May Mediate Brain Region Vulnerability to AD

“Epicenters” have been described as brain regions showing the most significant pathological alterations, hypothesized to be the initial site of disease onset [7,8]. Stressed “nodes” are known as brain regions with high network traffic, also referred to as “hubs”, that experience activity-induced deterioration that can lead to or exacerbate diseases [10,58]. While both concepts are instrumental in theories of AD etiology devised by connectome and network-based studies [7,8,10,58], the cell types underlying these “epicenters” and stressed “nodes” are unclear. Based on the regions that display reduced ADNP expression with aging, we propose that AHNP neurons serve as one of the cellular components of the “hubs” and “epicenters” leading to the onset and/or progression of AD. To test this, we analyzed the distribution of AHNP neurons across various regions of the human brain. Two potential observations and implications exist: (1) AHNP neurons ubiquitously exist in all brain regions, but those in early-AD-impacted regions are, regardless of the underlying cause, more susceptible to dysfunction than others, or (2) AHNP neurons are preferentially distributed in early-AD-impacted brain regions to physiologically perform cognitive functions, but they are more prone to dysfunction than other cell types, therefore mediating the regional vulnerability to AD with aging-related cell dysfunctions. We predict that a single-cell transcriptomic survey of brain regions would show that AHNP neurons are more abundantly distributed in early-AD-affected brain regions that engage extensively in memory and executive functions, such as the entorhinal cortex, hippocampus, and basal forebrain [59,60,61]. However, if the first scenario were true, our hypothesis could be negated altogether.

**Figure 5:**
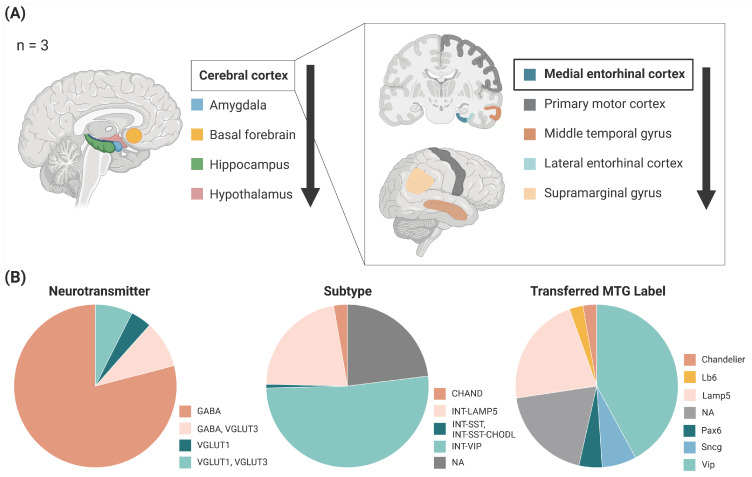
Local density of neurons co-expressing high levels of Alzheimer’s disease-associated neuropeptides (AHNP neurons) may govern brain vulnerability to AD. (**A**) Top five brain and cortical regions ranked by AHNP neuron abundance are visualized. n, sample size. Arrows indicate the decrease of abundance. (**B**) Categorization of AHNP neurons predominantly found in EC by neurotransmitter, subtype, and transferred MTG label (common cell type nomenclatures for the medial temporal gyrus of the mammalian brain) [12].

A recent study published single-cell transcriptome data from ~100 dissections across the forebrain, midbrain, and hindbrain of human donors and classified brain cells into 461 clusters [12]. The authors also compared their cell clusters with previous publications that used NP diversity to classify neurons [12,44]. We first investigated the top brain regions where AHNP neurons existed using cluster annotations provided by Siletti et al. and calculated the number of neurons in each of the top three brain regions and microdissections (see Section 2) [12]. As anticipated, the amygdala and hippocampus—where the age-associated decrease of ADNP expressions was observed [19]—were among the top five brain regions (Figure 5A; Table 2). While previous studies identified the hypothalamus as considerably relevant in AD [62,63], the hypothalamus did not show an age-related decrease in ADNPs. We also found that the cerebral cortex contained the most AHNP neurons, but this was likely due to the number of dissections assigned to the cerebral cortex. To overcome this issue, we set out to identify the distribution of AHNP neurons among cortical regions separately by ranking the cortical dissections based on the number of AHNP neurons. Again, we found that medial and lateral EC (MEC and LEC) ranked among the top five cortical regions (Figure 5A; Table 3). In particular, the MEC harbored the highest number of AHNP neurons among all micro-dissected regions (Table S13). In contrast, very few AHNP cells were found in the cerebellum, spinal cord, and medulla, which are generally thought to be spared by AD neuropathology (Table S14), further supporting our hypothesis that dysfunction of AHNP cells mediates the brain region-specific vulnerability to AD.

As the EC—the earliest and most heavily affected region in AD neuropathology—harbored the most significant population of AHNP neurons, we sought to investigate the specific cell types, as previous investigation has suggested that most ADNP co-expressing neurons are GABA-ergic [19]. Specifically, we aimed to understand how they align with established neuronal cell types. We selected neurons that had MEC in the top three dissections and examined the proportions of neuron types, including based on the neurotransmitter, subtypes, and transferred MTG labels (common cell type nomenclatures for the MTG regions of the mammalian brain) [12]. We found that most of the AHNP neurons were indeed GABA-ergic interneurons; however, about a quarter of the neurons have not been described before (Figure 5B).

## 4. Discussion

Why some ubiquitously expressed proteins (e.g., tau and Aβ) exhibit selective accumulation in particular regions of the brain and cells, yet spare their comparable neighbors, is a fundamental question of AD research. Examining multiscale and spatiotemporal RNA-seq data from 1890 human brain samples, we aimed to gain a comprehensive understanding of the potential mechanistic roles that NP-intensive neurons that co-express high levels of NPs (HNP neurons) play in mediating the selective vulnerability of brains to AD.

To address our previous hypothesis that HNP neurons, given their secretory and peptidal signaling functions, would demand more translation and transportation, leading to increased metabolic vulnerability [19], we investigated the specific characteristics of HNP neurons and the link between HNP neuron dysfunction and the hallmark molecular indicators of AD. We note that the criteria for HNP neurons in this study were primarily based on the co-expression of AD-associated NPs (ADNPs), which may be subject to change with advancements in sequencing technology, the availability of more datasets, and the discovery of additional NPs. Due to the significant variability in the proportion of neurons expressing NPs across different datasets (see Section 2), we utilized the NP list compiled in our previous study to maintain consistency in the follow-up analysis on the Grubman dataset [19,25]. To facilitate the incorporation of more NPs and additional datasets as more data are generated and datasets with better NP characterization become available, we have made the source code used in this study available for reanalysis. In addition, using co-expressed ADNPs to label neuronal subpopulations was of timely value for AD research because many neuronal subpopulations have complex combinatorial expression of NPs, and many of them exist outside of characterized cell populations [12,64].

Acknowledging these complexities, we found that (1) HNP neurons were more metabolically active and had gene expression profiles suggesting higher connectivity; (2) MEC AD neurons co-expressing higher levels of NPs showed the molecular signature of AD, including protein misfolding; and (3) the deficiency of AD cells was linked to loss of function of HNP cells. While we anticipated a greater metabolic burden as a source of neuronal vulnerability for HNP neurons, it was surprising to discover that HNP neurons can be predisposed to tau hyperphosphorylation and misfolding [49,65]. In addition to *ERBB4*, recent research has revealed that the impairment of NMD, an elevated process observed in HNP cells and suppressed by cellular stress, mediates tau-induced neurotoxicity [66]. Therefore, we posit that the disruption of cellular processes elevated in HNP neurons could occur more readily/earlier in these cells and contribute to the formation of misfolded proteins within them, subsequently leading to their selective degeneration.

To test the idea that the loss of ADNPs expressed by HNP neurons could participate in early AD development and/or progression, we considered the perspectives of neuropathology, cognitive status, and spatial progression during aging. Although we anticipated a decrease in ADNP expression with age and AD progression, it was surprising to observe this phenomenon in donors with neuropathological changes as early as Braak stage 2. While the majority of MCI donors in the MIT ROSMAP Multiomics dataset presented neuropathology falling in Braak stages 1 and 2, deviating from the typical distribution where most MCI patients fall between stages 3–4 [67,68], the significant decline in HNP neuron abundance among MCI brains in this dataset further substantiates the early alteration of ADNP expressions associated with AD progression. Despite both datasets being unsuitable for the functional characterization of HNP neurons (as detailed in the Section 2), our findings indicate that the ADNPs identified in the Grubman dataset, when analyzed alongside other single-cell sequencing datasets, highlight HNP neurons relevant to Alzheimer’s disease (AD). This suggests that the loss of ADNPs may represent an early molecular alteration in the brains of individuals at risk for developing AD. We note that while the disproportionate absence of HNP/ADNP co-expressing HNP (AHNP) neurons was described as selective degeneration in this manuscript, with the increased proportion of cells not expressing any NPs observed in multiple datasets, the degeneration could be interpreted as neuronal death and/or loss of functions. The definitive interpretation will need longitudinal tracking of neurodegeneration at the single-neuron level.

If AHNP neurons were indeed important for early AD, their presence in physiological conditions should help explain the regional vulnerability seen in AD. The preferential localization of AHNP neurons in regions of the brain that are susceptible to AD, such as the medial entorhinal cortex, amygdala, basal forebrain, and hippocampus, further underscores their potential role as key cellular contributors to the disease’s pathology. Two brain regions from the analyses were surprising to us—the hypothalamus and primary cortex (M1). Firstly, while the hypothalamus was highlighted in the regional distribution of AHNP neurons and demonstrated atrophy in AD [69], its expression of ADNPs did not decrease significantly with age. This difference may be attributed to AD-specific changes in hypothalamus being more disease-specific than age-related. The initial surprise at finding AHNP neurons concentrated in the M1 stems from the fact that motor deficits typically manifest later in AD [70]. However, the absence of overt motor symptoms in AD can be attributed to compensatory neural rewiring and hyperexcitability in the motor regions, rather than the non-existence of neuropathology [71,72]—this is not mutually exclusive with the proposed selective degeneration, as the term could be interpreted as loss of function rather than cell death, as discussed above. Notably, variant AD with abnormal tau accumulation in the M1 has been reported [73]. As AHNP neurons likely play a role in maintaining M1 microenvironment homeostasis, their dysfunction could exacerbate regional vulnerabilities, potentially contributing to upper motor neuron dysfunction and other neurodegenerative diseases, such as amyotrophic lateral sclerosis (ALS). The presence of neurofibrillary tangles in ALS [1,74] and the molecular signatures of AHNP neurons predisposing them to tau misfolding suggest that these neurons may be relevant in understanding mixed pathologies, common susceptibilities, and overlapping etiologies across neurodegenerative disorders. Considering the changes of AHNP neurons in MCI donors and the alterations in expression of ADNPs within cognitive regions during aging, an alternative to our hypothesis could be that these neurons increase the risk of cognitive decline and tau-related neurodegenerative diseases, rather than being AD-specific. While this perspective necessitates a re-evaluation of our original hypothesis, it may be of greater importance, as it could potentially address the prevention of a spectrum of neurodegenerative diseases, including AD.

Our findings contribute to the broader framework of AD theories and our understanding of the transition from brain function to dysfunction. Macroscopically, they support the “nodal stress” hypothesis [9,10,58], which posits that brain regions with strong anatomical connectivity are particularly vulnerable to damage due to their heightened susceptibility to cytotoxic events. This hypothesis aligns with the understanding that certain brain regions, which accumulate pathologically associated proteins, are also those that contain the most vulnerable cell types and are often the earliest affected in the disease’s progression [7,8,9]. Microscopically, HNP neurons with enhanced connectivity and high metabolic activity may be especially susceptible to the deleterious effects of cytotoxic events and the presence of misfolded proteins [3,58]. The observed decline in ADNP expression with age, coupled with the physiological distribution of HNP neurons in regions vulnerable to Alzheimer’s disease, suggests a critical link between macroscopic brain vulnerability and specific microscopic cellular components. This connection implies that while regional vulnerabilities can be observed at a larger scale, they may be driven by the dysfunction of particular cell types, such as HNP neurons. Furthermore, cellular dysfunctions, marked by distinct molecular pathways and biochemical properties, play a crucial role in the onset and progression of AD [9]. Extensive research over the past decade has illuminated the connectomic landscape of neurodegenerative diseases, enhancing our understanding of the brain regions and neural circuits that are susceptible to AD [7,8,58,75]. However, the specific cell types and cellular mechanisms that underpin these connectomic findings remain largely unexplored. Our findings potentially bridge this gap by highlighting the importance of HNP neurons in the context of AD, emphasizing their potential role in both the physiological and pathological processes that characterize the disease.

ADNPs are known to play important roles in cellular processes crucial to the pathogenesis of neurodegeneration, such as mitochondrial dysfunction, persistent neuroinflammation, and disrupted circadian rhythm (briefly summarized by Li and Larsen) [19]. Together with the mechanistic considerations, the alterations in the abundance and diversity of ADNP-producing neurons during different aspects of AD progression as well as the spatially specific reduced expression of ADNPs during aging identified in this study indicate that decreased expression of ADNPs by neurons may accelerate, or even drive, the progression of protein misfolding, cognitive decline, and neurodegeneration in AD.

Given the limited regenerative capacity of neurons, early detection and treatment of AD are paramount. Our research highlights the relevance of ADNPs as combinatorial and longitudinal biomarkers to evaluate the risk and progression of AD development. For instance, an analysis of the CSF proteome identified that CHGA and VGF exhibited significant differences in abundance among the CT, MCI, and AD groups [76]. As such, CSF levels of ADNPs could serve as direct biomarkers for AD. As aging progresses, peripheral tissues could also be valuable for tracking these changes. Specifically, monitoring ADNP levels in more accessible tissues such as blood, skin, or saliva could provide a less invasive method for longitudinal tracking of AD progression and response to treatments. Even though ADNPs may be secreted by other tissue types, there could be proxy peripheral blood biomarkers reflecting changes of ADNPs in the brain. This potential connection can be explored through longitudinal studies correlating CSF ADNP levels with blood biomarker identification, such as gene expression panels. Such an approach could lead to the development of less invasive and more accessible diagnostic and monitoring tools for AD. Additionally, further studies validating these findings and investigating the underlying mechanisms responsible for the observed decline in AHNP neuron abundance are needed. Evaluating whether interventions aimed at mimicking and/or preserving the ADNP signaling network could impede the progression of AD is also valuable, especially considering the subtle differences in GPCR expression observed between CT and AD [19]. Collectively, these observations suggest that deficiencies in ADNPs with aging contribute to AD development and progression and that these deficits could be a consequence of losing AHNP neurons during aging, which underlies the shared cognitive decline during aging and in early AD.

It is crucial to acknowledge that the interpretation and generalizability of these results may be limited by the lack of diversity in the dataset. The sole dataset available for this analysis was derived exclusively from brain samples of white male individuals. It is well-established that gene expression varies both within and between different ethnic groups [41,77] and sexes [78,79,80]; future research collecting more inclusive datasets is needed to ensure that knowledge gained from data analysis benefits the wider population. As NPs and their signaling activities are highly influenced by sex hormones [78,79,80], such datasets could help understand the epidemiology of AD and the differential accumulation of tau proteins between sexes during the aging process [1,81]. Other potential confounding variables, such as transcription factors and epigenetic mechanisms, warrant careful consideration. Recent research has made tremendous advancements on providing a comprehensive multiomic brain atlas for physiological conditions [12,82,83]. Our findings underscore the importance of extending these efforts to investigate the molecular landscapes of brains in various disease states. By understanding how dysfunction in HNP neurons occurs with aging and its detrimental effects on interconnected behavioral domains, we can gain valuable insights into the cell types and dysfunctions at the early stages of AD to develop effective disease models as well as cell-type-specific targeting strategies for prevention and therapy.

## 5. Conclusions

NPs play essential roles in cellular communication and homeostasis but can confer metabolic burdens due to their synthesis. This study demonstrates that NP-intensive neurons display multifaceted properties associated with AD vulnerability: (1) characterized by heightened metabolic activity and susceptibility to tau hyperphosphorylation; (2) exhibiting disproportionate and site-specific depletion during early AD progression, manifesting in both cognitive and neuropathological alterations; and (3) showing spatial distribution that correlates with AD-vulnerable brain regions under physiological conditions. We conclude that NP-intensive neurons likely participate in AD development and early progression. While causality studies are warranted to substantiate these findings, this avenue of research is promising, as understanding the roles of NPs in neuronal and cellular vulnerability of AD could facilitate earlier detection and intervention.

## Figures and Tables

**Table 1:** Significantly decreased expression of ADNPs with aging only occurred in early AD-impacted regions.

Brain Region	Sample Size	Correlation	*p*-Value	Significance
Hippocampus	103	−0.30	0.00	*
Frontal cortex (BA9)	116	−0.34	0.00	*
Anterior cingulate cortex (BA24)	88	−0.30	0.01	*
Amygdala	73	−0.30	0.02	*
Hypothalamus	109	0.02	0.85	
Caudate basal ganglia	146	−0.08	0.28	
Nucleus accumbens basal ganglia	143	0.00	0.96	
Putamen basal ganglia	126	0.11	0.25	
Substantia nigra	66	−0.23	0.08	
Cerebellar hemisphere	141	−0.03	0.74	
Cerebellum	156	−0.08	0.34	

ADNP, Alzheimer’s disease (AD)-associated neuropeptides; BA, Brodmann area; *, *p*-value < 0.05. Conditional test for association [43], α = 0.05.

**Table 2:** Top five brain regions ranked by AHNP neuron abundance.

Brain Regions	AHNP Neuronal Count
Cerebral cortex	218,145
Amygdala	72,256
Basal forebrain	45,559
Hippocampus	40,870
Hypothalamus	23,217

**Table 3:** Top five cortical regions ranked by AHNP neuron abundance.

Cortical Regions	Full Cortical Description	AHNP Neuronal Count
MEC	Anterior parahippocampal gyrus, posterior part (APH)—Medial entorhinal cortex	19,206
M1C	Precentral gyrus (PrCG)—Primary motor cortex	11,821
MTG	Middle Temporal Gyrus	10,654
LEC	Anterior parahippocampal gyrus (AG)—Lateral entorhinal cortex	2625
A40	Supramarginal gyrus (SMG)—A40	1732

## Data Availability

Datasets utilized in this manuscript are publicly available as stated in the availability of data and materials. Three publicly available single-cell RNA-sequencing datasets of the human EC were included for analysis in this study: Grubman et al. (http://adsn.ddnetbio.com, accessed on 31 May 2022), Leng et al. (https://cellxgene.cziscience.com/datasets, accessed on 21 May 2024), and the MIT ROSMAP Single-Nucleus Multiomics Study (https://adknowledgeportal.synapse.org/Explore/Studies/DetailsPage/StudyDetails?Study=syn52293417, accessed on 2 October 2023). GTEx v8 data can be downloaded from the GTEx portal (https://gtexportal.org/home/downloads/adult-gtex/bulk_tissue_expression, accessed on 3 October 2022). Single-cell sequencing of microdissected brain regions by Siletti et al. can be found on GitHub (https://github.com/linnarsson-lab/adult-human-brain, accessed on 18 May 2024). Code to reproduce findings can be found on GitHub (https://github.com/mancili/HNP/).

## Supplementary Materials

The following supporting information can be downloaded at: https://www.mdpi.com/article/10.3390/biom14121518/s1, Figure S1: Neurons from Leng and MIT ROSMAP Multiomics datasets showed insufficient neuronal populations expressing neuropeptides (NPs); Figure S2: The transcript level of neuropeptides (NPs) is highly correlated with the number of co-expressed NPs; Figure S3: Disproportionate absence of neurons in the high neuropeptides (NPs)-producing group where 6+ NPs are co-expressed; Figure S4: *ERBB4* expression shares a significant positive correlation with the number of co-expressed neuropeptides (NPs) in neurons; Table S1: List of Alzheimer’s disease-associated neuropeptides (ADNPs); Table S2: De-identified metadata for individuals and experiments included from the MIT ROSMAP Multiomics dataset; Table S3: De-identified metadata for individuals and experiments included from the GTEx dataset; Table S4: Cell count for different cell types co-expressing various levels of neuropeptides (NP) in Alzheimer’s disease (AD) and control (ct) donor brains from the Grubman dataset; Table S5: The molecular process increased in control HNP neurons in comparison to control LNP neurons; Table S6: Genes with significantly increased expression in HNP neurons; Table S7: Summarization of generalized linear regression analysis of gene expression and number of co-expressed neuropeptides (NPs); Table S8: Genes showing significantly decreased expression in AD MNP neurons; Table S9: Molecular process increased in Alzheimer’s disease MNP neurons in comparison to control MNP neurons; Table S10: Alzheimer’s disease-associated neuropeptides (ADNPs) show decreased expression with aging in early AD-impacted regions; Table S11: The expression of neuropeptides (NPs) that are not Alzheimer’s disease-associated NPs (ADNP) across brain regions with aging are not specific to early AD regions; Table S12: Neuropeptide (NP) expression and their change with age in each brain region; Table S13: Distribution of AHNP neurons across micro-dissected brain regions; Table S14: Distribution of AHNP neurons across brain regions.
