# Supplementary material for: Multifaceted Role of Specialized Neuropeptide-Intensive Neurons on the Selective Vulnerability to Alzheimer’s Disease in the Human Brain
Source: Biomolecules. 2024 Nov 27;14(12):1518. doi: 10.3390/biom14121518 (PMC11673071; doi:10.3390/biom14121518)
Supplement: Supplementary file 1 [file biomolecules-14-01518-s001.zip › Supplementary Materials.pdf]

--Supplementary Materials

1. Supplementary Tables

Table S1: List of Alzheimer's disease-associated neuropeptides (ADNPs). See Li & Larsen (2023) for details.[1]

Table S2. De-identified metadata for individuals and experiments included from the MIT ROSMAP Multiomics dataset. Barcode, cell ID; EC, entorhinal cortex; projid, de-identified donor ID; Exc, excitatory. Refer to the ADKnowledge portal for detailed information on experimental design and assay.[2]

Table S3. De-identified metadata for individuals and experiments included from the GTEx dataset. SUBJID, de-identified donor ID; DTHHRDY, death classification based on the 4-point Hardy Scale. See GTEx publications for additional details.[3]

Table S4. Cell count for different cell types co-expressing various levels of neuropeptides (NP) in Alzheimer's disease (AD) and control (ct) donor brains from the Grubman dataset. Neu, neuron; ast, astrocyte; end, endothelial cell; mic, microglia; opc, oligodendrocyte precursor cell; oli, oligodendrocyte; low, expressing 0-1 NP; mid, expressing 2-5 NPs; high, expressing 6+ NPs. See Li and Larsen (2023) for details.[1,4]

Table S5. The molecular process increased in control HNP neurons in comparison to control LNP neurons. HNP neurons, neurons expressing high levels of NPs, proxied by co-expressing 6+ NPs; LNP neurons, neurons expressing low levels of NPs, proxied by co-expressing 0-1 NP.

Table S6. Genes with significantly increased expression in HNP neurons. HNP neurons, neurons expressing high levels of NPs, proxied by co-expressing 6+ NPs.

Table S7. Summarization of generalized linear regression analysis of gene expression and number of co-expressed neuropeptides (NPs). A generalized linear regression model was fitted for genes that had significantly increased expression in neurons expressing high levels of NPs (See Table 5).

Table S8. Genes showing significantly decreased expression in AD MNP neurons. MNP, neurons expressing medium levels (co-expressing 2-5) of NPs.

Table S9. Molecular process increased in Alzheimer's disease MNP neurons in comparison to control MNP neurons. MNP neurons, neurons expressing medium levels of NPs, proxied by co-expression of 2-5 NPs.

Table S10. Alzheimer's disease-associated neuropeptides (ADNPs) show decreased expression with aging in early AD-impacted regions. cor_estimate, estimate of correlation; *****, p_value<0.05, α = 0.05.

Table S11: The expression of neuropeptides (NPs) that are not Alzheimer's disease-associated NPs (ADNP) across brain regions with aging are not specific to early AD regions. cor_estimate, estimate of correlation; *****, p_value<0.05, α = 0.05.

Table S12. Neuropeptide (NP) expression and their change with age in each brain region. RDS file of NP expression in brain regions included in this study. Row names, de-identified donor ID; DTHHRDY, death classification based on the 4-point Hardy Scale; SUM, cumulative expression of transcripts (log-transformed transcript per million counts).

Table S13: Distribution of AHNP neurons across micro-dissected brain regions. AHNP neurons, neurons co-expressing high levels of Alzheimer’s disease-associated neuropeptides (ADNPs). See https://github.com/linnarsson-lab/adult-human-brain/blob/main/tables/dissections.xlsx for abbreviations.

Table S14: Distribution of AHNP neurons across brain regions. AHNP neurons, neurons co-expressing high levels of Alzheimer’s disease-associated neuropeptides (ADNPs).

2. Supplementary Figures

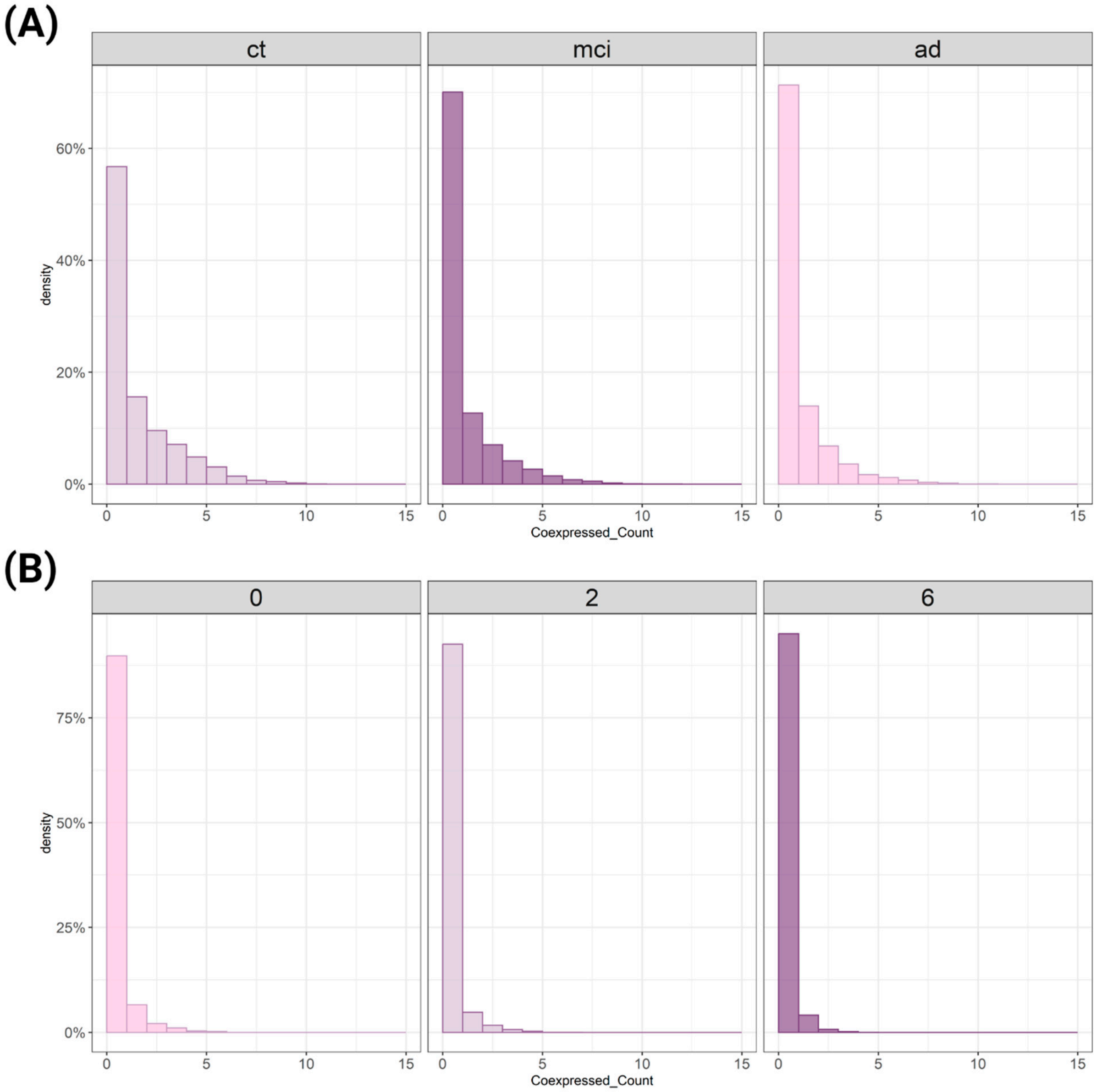

Figure S1. Neurons from Leng and MIT ROSMAP Multiomics datasets showed insufficient neuronal populations expressing neuropeptides (NPs) (A) Distribution of neurons based on the number of co-expressed NPs from the MIT ROSMAP Multiomics dataset. **(B)** Distribution of neurons based on the number of co-expressed NPs from the Leng dataset. ct, control; mci, mild cognitive impairment; ad, Alzheimer's disease; 0, Braak stage 0; 2, Braak stage 2; 6, Braak stage 6. Coexpressed_count, number of co-expressed NPs.

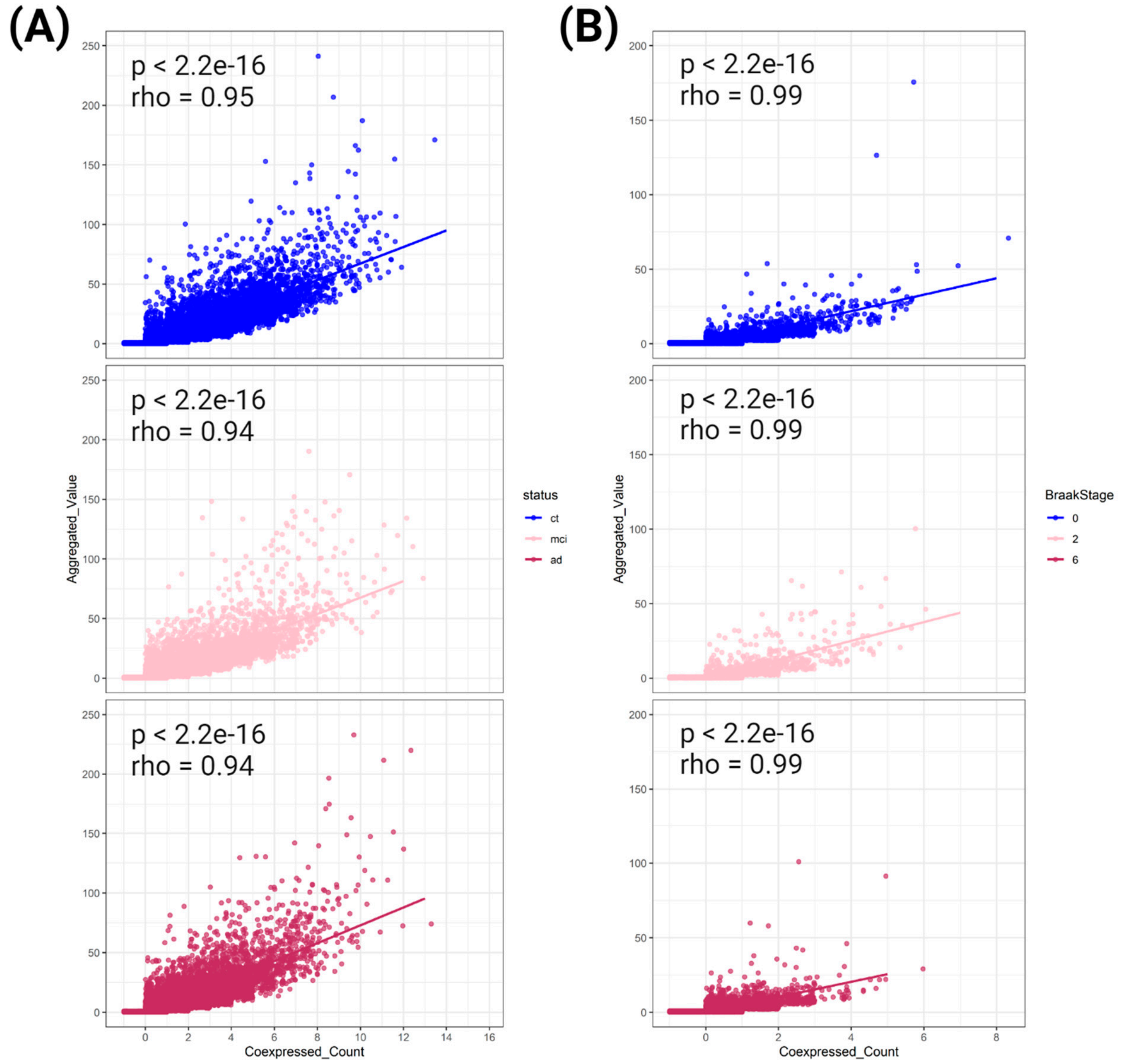

Figure S2. The transcript level of neuropeptides (NPs) is highly correlated with the number of co-expressed NPs. (A) Scatterplot showing the relationship between normalized transcript count and number of co-expressed NPs in neurons from the MIT ROSMAP Multiomics dataset. **(B)** Scatterplot showing the relationship between normalized transcript count and number of co-expressed NPs in neurons from the Leng dataset. The Spearman rank correlation test was used to calculate correlation and p-value. The correlation coefficient (rho) and p-values were reported. The significance cut-off was set at 0.05. ct, control; mci, mild cognitive impairment; ad, Alzheimer's disease; 0, Braak stage 0; 2, Braak stage 2; 6, Braak stage 6.

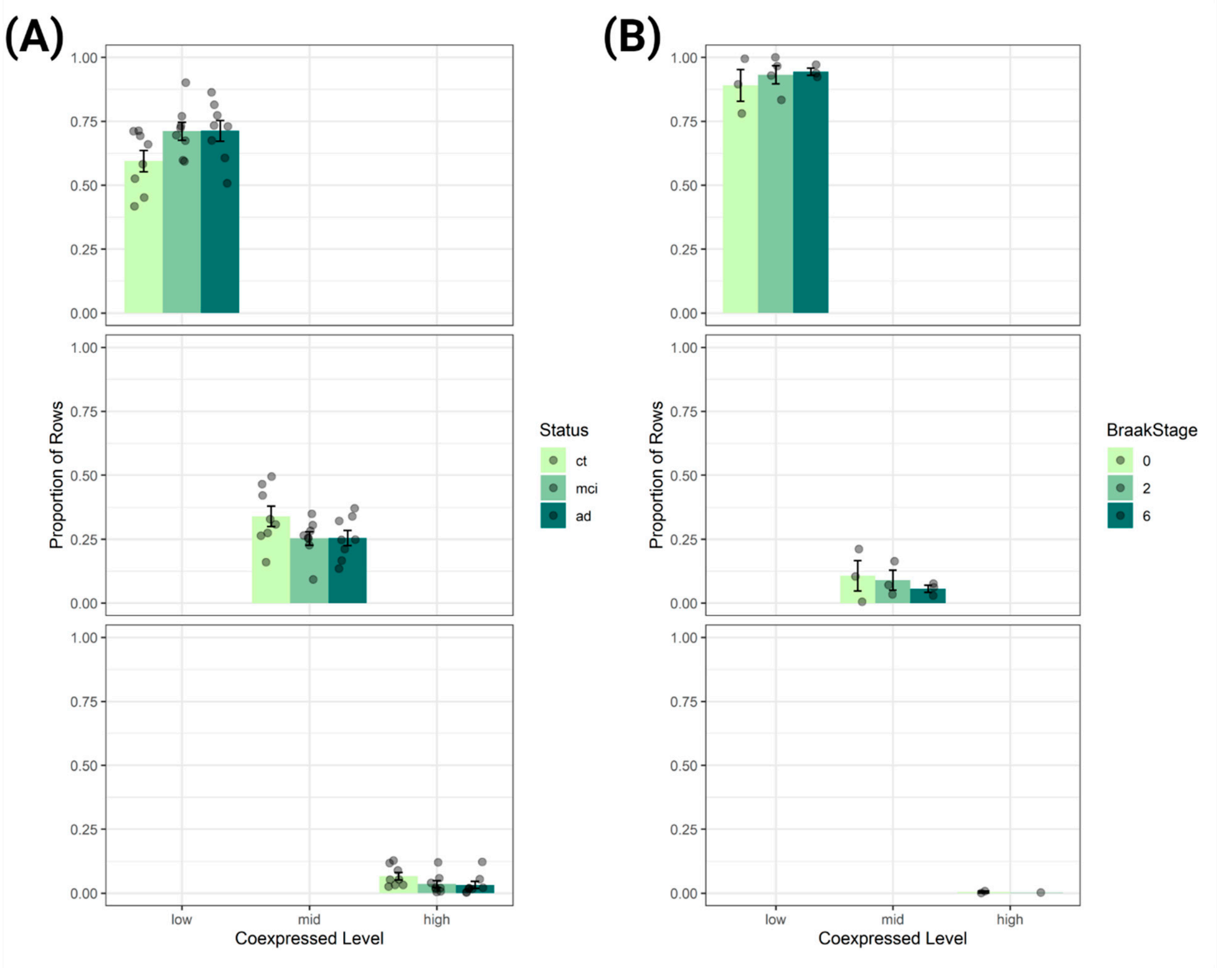

Figure S3. Disproportionate absence of neurons in the high neuropeptides (NPs)-producing group where 6+ NPs are co-expressed. (A) The distribution of neurons is based on the number of co-expressed NPs from the MIT ROSMAP Multiomics dataset. **B)** The distribution of neurons based on the number of co-expressed NPs from the Leng ROSMAP Multiomics dataset. Note the complete absence of HNP neurons in Braak stage 6. low: 0-1; middle (mid): 2-5, and high: 6+. ct, control; mci, mild cognitive impairment; ad, Alzheimer's disease; 0, Braak stage 0; 2, Braak stage 2; 6, Braak stage 6; HNP, neurons expressing high levels of NPs, proxied by co-expressing 6+ NP.

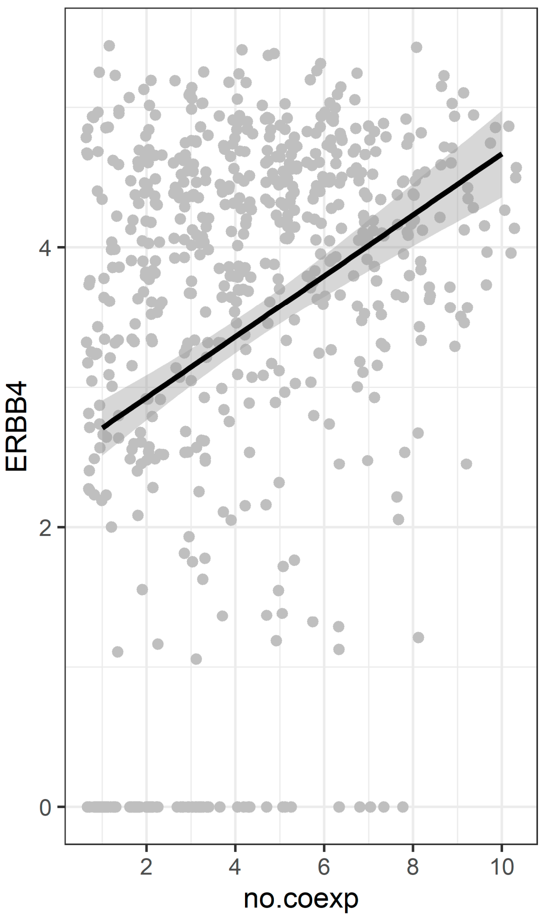

Figure S4. *ERBB4* expression shares a significant positive correlation with the number of co-expressed neuropeptides (NPs) in neurons. Scatterplot showing the relationship between normalized transcript count of *ERBB4* and number of co-expressed NPs in neurons from the Grubman dataset. No.coexp, number of co-expressed NPs.

3. Code used for bioinformatic analysis

See https://github.com/mancili/HNP/

Reference:

1. Li M, Larsen PA. Single-cell sequencing of entorhinal cortex reveals widespread disruption of neuropeptide networks in Alzheimer's disease. *Alzheimers Dement*. Published online February 24, 2023. doi:10.1002/alz.12979

2. AD knowledge portal. Accessed June 27, 2024. https://adknowledgeportal.synapse.org/Explore/Studies/DetailsPage/StudyDetails?Study=syn52293417

3. GTEx Consortium. The GTEx Consortium atlas of genetic regulatory effects across human tissues. *Science*. 2020;369(6509):1318-1330.

4. Grubman A, Chew G, Ouyang JF, et al. A single-cell atlas of entorhinal cortex from individuals with Alzheimer's disease reveals cell-type-specific gene expression regulation. *Nat Neurosci*. 2019;22(12):2087-2097.
